# The association of depression with lower urinary tract symptoms: data from the National Health and Nutrition Examination Survey, 2005–2008

**DOI:** 10.7717/peerj.7795

**Published:** 2019-10-01

**Authors:** Jee Soo Park, Won Sik Ham, Chang Hee Hong, Byung Ha Chung, Kyo Chul Koo

**Affiliations:** Department of Urology, Yonsei University College of Medicine, Seoul, Republic of Korea

**Keywords:** Lower urinary tract symptoms, Depression, NHANES

## Abstract

**Background:**

To identify the factors associated with lower urinary tract symptoms (LUTS), we investigated associations between psychological factors, including depression and sleep disorders, and LUTS using the National Health and Nutrition Examination Survey (NHANES) database.

**Materials and Methods:**

The NHANES database was examined for the period of 2005 to 2008. Men older than 40 years, who had completed questionnaires surveying their kidney/urologic, prostate, mental health, and sleep conditions were included in this study. LUTS was defined as the presence of two or more of the following symptoms: incomplete emptying, urinary hesitancy, urinary frequency, and nocturia. Multivariable models using logistic regression were used to compare groups of men with or without LUTS.

**Results:**

Of 1,820 participants, 110 (6.1%) men reported depression, and 235 (12.9%) presented with LUTS. Men with LUTS were older and had a significantly higher prevalence of depression and unemployment. Sleep disorder was not associated with LUTS. Multivariable logistic regression models demonstrated that men reporting moderate depression had the highest age-adjusted odds (odds ratio = 5.89, 95% CI [3.44–10.11]; *p* < 0.001) of reporting clinical LUTS.

**Conclusions:**

A significant association was observed between LUTS and depression, and between LUTS and employment status. Although the pathophysiology of these relationships is unclear, physicians should consider multi-disciplinary evaluation and treatment approaches for LUTS.

## Introduction

Lower urinary tract symptoms (LUTS) are common in aging men, with approximately 80.0% of men experiencing at least one urinary symptom by the age of 80 (Wei, Calhoun & Jacobsen, 2005). Treatment of LUTS is a substantial financial burden on the United States healthcare system (Hu et al., 2004). Moreover, LUTS has been found to have a significant negative impact on quality of life in several studies (Coyne et al., 2009; Robertson et al., 2007). Although LUTS is highly prevalent, poses an economic burden, and adversely affects quality of life, few treatment modalities for it exist, and although pharmacologic interventions are considered first-line therapy, Cindolo et al. (2015) reported that only 29.0% of patients use these drugs for 1 year, indicating that adherence to these medications is poor.

Most medications for LUTS focus on solving bladder outlet obstruction resulting from hypertrophy of the prostate. However, there are multifactorial causes of LUTS development, and the often reported ineffectiveness of pharmacologic therapies implies that the cause of LUTS is not always prostate-centric (Kakizaki & Koyanagi, 2000). Indeed, a variety of factors have been reported to be associated with the development and progression of LUTS, including age-dependent structural and functional changes in the bladder and urethra, neurological and hormonal changes, diabetes and obesity, psychological and behavioral changes, and alterations in sleep patterns (Kakizaki & Koyanagi, 2000; Helf et al., 2011; Helf et al., 2012; Tam, Helfand & Erickson, 2017).

Several studies have reported on associations between psychological factors and LUTS. Most studies have focused on depression, which has been widely reported to be associated with LUTS (Coyne et al., 2009; Hakkinen et al., 2008; Laumann et al., 2008; Steers, Litman & Rosen, 2008; Fitzgerald et al., 2007). A few have reported an association between sleep disorders and the development of nocturia. A recent study reported a relationship between sleep disorders and LUTS (Fantus et al., 2018).

To our knowledge, this is the first study to thoroughly investigate factors associated with LUTS from the National Health and Nutrition Examination Survey (NHANES), 2007–2008, which is a large, cross-sectional dataset that is representative of the United States population. We investigated demographic differences and psychological factors, such as depression and sleep disorders, in patients with LUTS in order to identify dominant factors associated with LUTS.

## Materials & Methods

### Data source

The NHANES is a cross-sectional observational study that collects health-related information using a complex, stratified, multistage, probability cluster design representative of the general non-institutionalized United States population. The Institutional Review Board of the National Center for Health Statistics approved the protocol, and all participants provided written informed consent to the National Center for Health Statistics. This study included datasets for 2005–2006 and 2007–2008, as only these datasets contained information related to both urinary symptoms and psychological factors including depression and sleep disorders.

### Study population

We included men aged 40 years or older who completed questionnaires surveying the following: kidney/urologic conditions, prostate conditions, mental health conditions, and sleep conditions. We excluded all men with prostate disease, including prostate cancer (15,839 patients), men with stroke who had comorbidities associated with neurogenic bladder (127 patients), and those with missing data (2,019 patients), leaving 1,820 eligible participants.

### LUTS

Several questions were used to assess LUTS including the following: (1) After urinating, does your bladder feel empty? (responses: yes/no, incomplete emptying); (2) Do you usually have trouble starting to urinate? (yes/no, hesitancy); (3) How often do you have urinary leakage? (defined as not able to hold urine until reaching a toilet at least once a month, urinary frequency); (4) How many times per night do you usually get up to urinate? (defined as waking at least twice per night to urinate, nocturia). Daytime LUTS was defined as the presence of more than one of the symptoms surveyed in questions 1–3. Clinical LUTS was defined as the presence of any two or more of the surveyed symptoms (questions 1–4).

### Depression

Depression was measured using the Nine-item Patient Health Questionnaire (PHQ-9) depression scale, a nine-item screener that asks questions about the frequency of symptoms of depression scored from 0 to 27, and that is widely used in both clinical and research settings (Kroenke & Spitzer, 2002). A PHQ-9 score of 10 or greater was used as a cutoff for identifying major depression (sensitivity 88.0% and specificity 88.0%) (Kroenke & Spitzer, 2002). Depression scores were categorized into four groups: minimal (<5), mild (5–9), moderate to moderately severe (10–14), and moderately severe to severe (≥15). The validity and reliability of PHQ and its nine-item depression module in depressive diagnosis and grade of severity have been widely documented (Kroenke, Spitzer & Williams, 2001; Kroenke et al., 2010; Spitzer, Kroenke & Williams, 1999; Spitzer et al., 2000).

### Other measurements

All participants were asked about age, race/ethnicity (non-Hispanic white, non-Hispanic black, Mexican American, or those who selected multiple races or other racial/ethnic groups), educational attainment (less than a high school diploma, high school graduate, or education beyond high school), household income (poverty income ratio [PIR], PIR <1, 1 ≤PIR<3, or PIR ≥3), married or living with a partner (yes or no), employment (yes or no), health insurance status (yes or no), smoking status (current, former, or never), and binge drinker (defined as having ever consumed ≥5 drinks of any kind of alcoholic beverage almost every day; yes or no). A prior self-reported history of coronary artery disease (CAD), congestive heart failure (CHF), chronic obstructive pulmonary disease (COPD), including emphysema and/or chronic bronchitis, and malignancies were included.

Hypertension was defined as the use of antihypertensive medication or a reported blood pressure of 140/90 mmHg or higher. Blood pressure was measured using a mercury sphygmomanometer. Diabetes was defined as a self-reported previous diagnosis of the disease by a healthcare provider or a hemoglobin A_1c_ level of 6.5% or greater (the diagnostic criterion for diabetes according to the American Diabetes Association 2010).

Height and weight were measured in a medical examination center, and body mass index (BMI) was calculated as weight in kilograms divided by height in meters squared. Participants were divided into three BMI groups: normal weight or underweight (BMI <25 kg/m^2^), overweight (BMI = 25 to <30 kg/m^2^), and obese (BMI ≥30 kg/m^2^).

A subject was considered to have a sleep disorder if they answered “yes” to the question “Have you been diagnosed as having a sleep disorder?”

### Statistical analysis

Data are reported as means ± standard deviations (SDs) for continuous variables and for categorical variables, data are reported as percentages. Since the size of the study population was large enough to represent the US population and the values were not skewed in clinical perspective, t-tests were used to compare continuous variables, and chi-square test was used to compare categorical variables in univariable analysis. Multivariable logistic regression models were used for multivariable analysis, including all risk factors that were significant in univariable analysis. Multicollinearity was measured using variance inflation factor (VIF) for 12 independent variables used in multivariable logistic regression models and VIFs were less than 2, showing that there was no problem with multicollinearity (DeMaris, 2004).

A model of multivariable logistic regression analysis was used to evaluate the association between the severity of depression and LUTS (clinical LUTS, daytime LUTS, and individual symptoms of incomplete bladder emptying, urinary hesitancy, urinary frequency, and nocturia) after adjusting for potential confounding factors. We evaluated the odds ratios (ORs) and 95% confidence intervals (CIs) of reporting clinical LUTS and individual symptoms after adjusting for potential confounders in two models: first, we added age and then we adjusted models for educational attainment, household income, employment, binge drinking, smoking status, hypertension, diabetes, CAD, CHF, COPD, and cancer. As LUTS is not common disease entity, we used ORs to estimate relative risks.

SPSS software, version 23.0 (SPSS Inc., Chicago, IL, USA) was used to conduct all statistical analyses, and all statistical tests were two-tailed. *P*-values <0.05 were considered statistically significant.

## Results

### Baseline characteristics

Baseline characteristics of the study population are shown in Table 1. The mean ±SD age of the participants was 56.9 ± 11.8 years. Their mean ±SD BMI was 28.9 ± 6.1 kg/m^2^, and 77.0% were included in the overweight or obese group. Overall, 30.0% of the participants had an educational level of less than a high school diploma, and 58.8% were currently working. Moreover, 25.1% were current smokers, and 27.4% were binge drinkers. The prevalences of hypertension and diabetes were 49.5% and 14.2%, respectively. In the 2005–2008 sample, 6.1% of participants had depression (PHQ-9 score ≥10). Among participants, 25.2% had daytime LUTS and 12.9% had clinical LUTS.

**Table 1:** Characteristics of the study population.

	Study population (*n* = 1,820)
Age (years)	56.9 ± 11.8
Race/ethnicity (%)	
Non-Hispanic white	968 (53.2%)
Non-Hispanic black	352 (19.3%)
Mexican American	316 (17.4%)
Other	184 (10.1%)
Height (cm)	174.4 ± 7.6
Weight (kg)	88.2 ± 20.2
BMI (kg/m^2^)	28.9 ± 6.1
BMI^a^ (%)	
Normal weight or underweight	419 (23.0%)
Overweight	773 (42.5%)
Obese	628 (34.5%)
SBP (mmHg)	129.6 ± 18.8
DBP (mmHg)	73.9 ± 13.2
Education <High school (%)	546 (30.0%)
Household income, PIR (%)	
PIR<1	289 (15.9%)
1 ≤PIR<3	713 (39.2%)
PIR ≥3	818 (44.9%)
Currently working (%)	1071 (58.8%)
Married or living with partner (%)	1325 (72.8%)
Insured (%)	1465 (80.5%)
Binge drinker^b^ (%)	498 (27.4%)
Cigarette smoking (%)	
Current	456 (25.1%)
Past	664 (36.5%)
Never	700 (38.5%)
Hypertension^c^ (%)	900 (49.5%)
Diabetes^d^ (%)	259 (14.2%)
CAD (%)	133 (7.3%)
CHF (%)	82 (4.5%)
COPD (%)	144 (7.9%)
Cancer (%)	129 (7.1%)
Sleep disorder (%)	158 (8.7%)
Depression (%)	
Mild (PHQ-9 Score of 5-9)	212 (11.6%)
Moderate (PHQ-9 Score of 10-14)	72 (4.0%)
Moderately Severe to Severe (PHQ-9 Score of ≥ 15)	38 (2.1%)
Daytime LUTS (%)	459 (25.2%)
Clinical LUTS (%)	235 (12.9%)

**Notes.**

Data are presented as mean ± SD for continuous variables and percentages for categorical variables.

SD standard deviation, BMI body mass index, CAD coronary artery disease, CHF congestive heart failure, COPD chronic obstructive pulmonary disease, DBP diastolic blood pressure, SBP systolic blood pressure, PHQ-9 9-item patient health questionnaire, PIR poverty to income ratio, LUTS lower urinary tract symptoms.

a BMI was divided into three groups: normal weight or underweight (BMI < 25 kg/m^2^), overweight (BMI = 25 to < 30 kg/m^2^), and obese (BMI ≥30 kg/m^2^).

b Defined as having ever consumed five drinks or more of any kind of alcoholic beverage almost every day.

c Defined as use of antihypertensive medication or a reported blood pressure ≥140/90 mmHg.

d Defined as self-reported diabetes diagnosed by a health professional (all adults) or a hemoglobin A_1c_ level ≥6.5%.

### Factors associated with daytime LUTS

Men with daytime LUTS were significantly older and had higher prevalences of cigarette smoking, hypertension, CAD, CHF, COPD, and depression, whereas their household income and employment level were significantly lower than those of men without LUTS (Table 2).

**Table 2:** Characteristics of the study population according to daytime and clinical LUTS.

	Daytime LUTS			Clinical LUTS		
	Daytime LUTS	No Daytime LUTS	P^e^	Clinical LUTS	No Clinical LUTS	P^e^
	(*n* = 459)	(*n* = 1,361)		(*n* = 235)	(*n* = 1,585)
Age (years)	59.7 ± 12.1	55.9 ± 11.6	<0.001	61.9 ± 12.2	56.1 ± 11.6	<0.001
Race/ethnicity (%)			0.707			0.331
Non-Hispanic white	249 (54.2%)	719 (52.8%)		131 (55.7%)	837 (52.8%)	
Non-Hispanic black	91 (19.8%)	261 (19.2%)		48 (20.4%)	304 (19.2%)	
Mexican American	79 (17.2%)	237 (17.4%)		40 (17.0%)	276 (17.4%)	
Other	40 (8.7%)	144 (10.6%)		16 (6.8%)	168 (10.6%)	
Height (cm)	174.2 ± 7.6	174.5 ± 7.6	0.487	174.2 ± 7.4	174.4 ± 7.6	0.704
Weight (kg)	88.3 ± 23.7	88.2 ± 18.9	0.934	89.1 ± 21.3	88.1 ± 20.0	0.512
BMI (kg/m^2^)	29.0 ± 7.5	28.9 ± 5.5	0.646	29.3 ± 6.3	28.9 ± 6.0	0.393
BMI (%)			0.625			0.310
Normal weight or underweight	113 (24.6%)	306 (22.5%)		58 (24.7%)	361 (22.8%)	
Overweight	189 (41.2%)	584 (42.9%)		89 (37.9%)	684 (43.2%)	
Obese	157 (34.2%)	471 (34.6%)		88 (37.4%)	540 (34.1%)	
SBP (mmHg)	130.5 ± 20.3	129.3 ± 18.2	0.275	132.0 ± 21.7	129.2 ± 18.3	0.062
DBP (mmHg)	73.3 ± 14.0	74.0 ± 13.0	0.323	73.1 ± 14.6	74.0 ± 13.0	0.365
Education <High school (%)	147 (32.0%)	399 (29.3%)	0.273	88 (37.4%)	458 (28.9%)	0.008
Household income, PIR (%)			0.023			<0.001
PIR<1	75 (16.3%)	214 (15.7%)		43 (18.3%)	246 (15.5%)	
1 ≤PIR<3	202 (44.0%)	511 (37.5%)		114 (48.5%)	599 (37.8%)	
PIR ≥3	182 (39.7%)	636 (46.7%)		78 (33.2%)	740 (46.7%)	
Currently working (%)	242 (52.7%)	829 (60.9%)	0.002	95 (40.4%)	976 (61.6%)	<0.001
Married or living with partner (%)	322 (70.2%)	1,003 (73.7%)	0.146	163 (69.4%)	1,162 (73.3%)	0.204
Insured (%)	377 (82.1%)	1,088 (79.9%)	0.305	194 (82.6%)	1,271 (80.2%)	0.393
Binge drinker (%)	137 (29.8%)	361 (26.5%)	0.167	78 (33.2%)	420 (26.5%)	0.032
Cigarette smoking (%)			0.027			0.001
Current	120 (26.1%)	336 (24.7%)		65 (27.7%)	391 (24.7%)	
Past	186 (40.5%)	478 (35.1%)		106 (45.1%)	558 (35.2%)	
Never	153 (33.3%)	547 (40.2%)		64 (27.2%)	636 (40.1%)	
Hypertension (%)	257 (56.0%)	643 (47.2%)	0.001	143 (60.9%)	757 (47.8%)	<0.001
Diabetes (%)	75 (16.3%)	184 (13.5%)	0.135	46 (19.6%)	213 (13.4%)	0.012
CAD (%)	46 (10.0%)	87 (6.4%)	0.010	26 (11.1%)	107 (6.8%)	0.018
CHF (%)	31 (6.8%)	51 (3.7%)	0.007	24 (10.2%)	58 (3.7%)	<0.001
COPD (%)	53 (11.5%)	91 (6.7%)	0.001	31 (13.2%)	113 (7.1%)	0.001
Cancer (%)	41 (8.9%)	88 (6.5%)	0.075	29 (12.3%)	100 (6.3%)	0.001
Sleep disorder (%)	42 (9.2%)	116 (8.5%)	0.680	23 (9.8%)	135 (8.5%)	0.519
Depression (%)			<0.001			<0.001
Mild (PHQ-9 Score of 5–9)	75 (16.3%)	137 (10.1%)		50 (21.3%)	162 (10.2%)	
Moderate (PHQ-9 Score of 10–14)	36 (7.8%)	36 (2.6%)		24 (10.2%)	48 (3.0%)	
Moderately Severe to Severe (PHQ-9 Score of ≥ 15)	15 (3.3%)	23 (1.7%)		10 (4.3%)	28 (1.8%)	

**Notes.**

Data are presented as mean ± SD for continuous variables and percentages for categorical variables.

BMI body mass index, CAD coronary artery disease, CHF congestive heart failure, CI confidence interval, COPD chronic obstructive pulmonary disease, DBP diastolic blood pressure, OR odds ratio, SBP systolic blood pressure, PHQ-9 9-item patient health questionnaire, PIR poverty to income ratio, LUTS lower urinary tract symptoms.

a BMI was divided into three groups: normal weight or underweight (BMI <25 kg/m ^2^), overweight (BMI = 25 to <30 kg/m ^2^), and obese (BMI ≤30 kg/m ^2^).

b Defined as having ever consumed five drinks or more of any kind of alcoholic beverage almost every day.

c Defined as use of antihypertensive medication or a reported blood pressure ≥140/90 mmHg.

d Defined as self-reported diabetes diagnosed by a health professional (all adults) or a hemoglobin A_1c_ level ≥6.5%.

e *P*-value calculated using the *t*-test (continuous data) or chi-square test (categorical data).

### Factors associated with clinical LUTS

The characteristics of men with and without clinical LUTS were also compared (Table 2). Age, household income, employment status, cigarette smoking, hypertension, CAD, CHF, COPD, depression, educational attainment, binge drinking, diabetes, and cancer were significantly associated with clinical LUTS in univariable analysis.

### Association between PHQ-9 score and clinical LUTS

Logistic regression analyses were performed to examine the association between LUTS and depression (Table 3). With clinical LUTS (two or more symptoms) as the outcome, participants reporting moderate to severe depression had the highest ORs of those reporting LUTS. In the age-adjusted model, moderate to severe depression was significantly associated with clinical LUTS (OR = 5.05, 95% CI [3.20–7.97]). Similar results were observed (OR = 4.10, 95% CI [2.50–6.70]) in the multivariable adjusted model, which was adjusted for factors that were significantly different between men with and without clinical LUTS. When examining individual LUTS (incomplete emptying, urinary hesitancy, urinary frequency, and nocturia), depression was associated with increased risk of each LUTS. Participants with major depression (PHQ-9 score ≥10) were further divided into two groups: (1) moderate depression and (2) moderately severe to severe depression. The logistic regression model showed that the moderate depression group showed higher odds (OR = 5.89, 95% CI [3.44–10.11]) with clinical LUTS, and individual LUTSs, except incomplete emptying, than the moderately severe to severe depression group (OR = 3.74, 95% CI [1.75–8.00]).

**Table 3:** Association between the PHQ-9 score and LUTS.

	PHQ-9 Categories					
	Minimal	Mild	Moderate to severe^a^		Moderate	Moderately severe to severe
	0–4	5–9	10–27	*p*^b^	10–14	15–27
	(*n* = 1,498)	(*n* = 212)	(*n* = 110)		(*n* = 72)	(*n* = 38)
Clinical LUTS (2 or more symptoms):^c^	151 (10.1%)	50 (23.6%)	34 (30.9%)	*p* < 0.001	24 (33.3%)	10 (26.3%)
Age adjusted OR (95% CI)^d^	1.00	3.27 (2.25–4.76)	5.05 (3.20–7.97)		5.89 (3.44–10.11)	3.74 (1.75–8.00)
Multivariate adjusted OR (95% CI)^e^	1.00	2.93 (1.99–4.30)	4.10 (2.50–6.70)		4.71 (2.67–8.32)	3.07 (1.39–6.81)
Daytime LUTS	333 (22.2%)	75 (35.4%)	51 (46.4%)	*p* < 0.001	36 (50.0%)	15 (39.5%)
Age adjusted OR (95% CI)^d^	1.00	2.12 (1.55–2.89)	3.44 (2.30–5.14)		2.47 (1.26–4.82)	1.03 (1.02–1.04)
Multivariate adjusted OR (95% CI)^e^	1.00	2.11 (1.53–2.91)	3.41 (2.22–5.24)		3.95 (2.37–6.57)	2.54 (1.26–5.12)
Incomplete emptying:	119 (7.9%)	29 (13.7%)	18 (16.4%)	*p* = 0.002	11 (15.3%)	7 (18.4%)
Age adjusted OR (95% CI)^d^	1.00	1.94 (1.25–3.00)	2.44 (1.41–4.19)		2.28 (1.16–4.46)	2.74 (1.18–6.37)
Multivariate adjusted OR (95% CI)^e^	1.00	1.76 (1.12–2.76)	1.92 (1.06–3.47)		1.75 (0.85–3.57)	2.28 (0.94–5.55)
Urinary hesitancy:	82 (5.5%)	23 (10.8%)	12 (10.9%)	*p* = 0.003	9 (12.5%)	3 (7.9%)
Age adjusted OR (95% CI)^d^	1.00	2.32 (1.42–3.81)	2.43 (1.27–4.64)		2.91 (1.38–6.13)	1.62 (0.48–5.40)
Multivariate adjusted OR (95% CI)^e^	1.00	2.58 (1.55–4.30)	3.37 (1.67–6.79)		3.87 (1.75–8.55)	2.43 (0.70–8.45)
Urinary frequency:	183 (12.2%)	40 (18.9%)	33 (30.0%)	*p* < 0.001	23 (31.9%)	10(26.3%)
Age adjusted OR (95% CI)^d^	1.00	1.83 (1.25–2.68)	3.49 (2.24–5.44)		3.92 (2.31–6.65)	2.78 (1.32–5.86)
Multivariate adjusted OR (95% CI)^e^	1.00	1.85 (1.25–2.74)	3.50 (2.16–5.69)		3.81 (2.17–6.68)	2.94 (1.33–6.51)
Nocturia:	403 (26.9%)	98 (46.2%)	55 (50.0%)	*p* < 0.001	37 (51.4%)	18 (47.4%)
Age adjusted OR (95% CI)^d^	1.00	2.94 (2.15–4.02)	3.55 (2.36–5.34)		3.93 (2.39–6.46)	2.93 (1.50–5.72)
Multivariate adjusted OR (95% CI)^e^	1.00	2.64 (1.91–3.65)	2.84 (1.84–4.41)		3.18 (1.89–5.36)	2.29 (1.14–4.62)

**Notes.**

CI confidence interval, OR odds ratio, LUTS lower urinary tract symptoms, PHQ-9 9-item Patient Health Questionnaire.

a Moderate to severe group for which a PHQ-9 score ≥10 was defined as the major depression group.

b Differences of prevalence of LUTS between minimal, mild, and moderate to severe group was analyzed using the chi-square test.

b Reported as at least 2 of 4 LUTS (incomplete bladder emptying, trouble starting to urinate, urinary frequency, and nocturia defined as 2 or more voids per night). This analysis included only those men with no clinical LUTS or 2 or more LUTS reported.

c Adjusted for age in years.

d Adjusted for age in years, educational attainment, household income, employment, binge drinking, smoking status, hypertension, diabetes, coronary artery disease, congestive heart failure, chronic obstructive pulmonary disease, and cancer.

## Discussion

In our study, daytime LUTS and clinical LUTS were significantly associated with age, employment status, and depression. We also found that men with greater depression scores were more likely to have LUTS, with the exception of those in the most severe depression group. We modeled LUTS evaluated its association with severity of depression. While treatment of LUTS in men has been mainly prostate-centric, our study suggests that multiple factors might be involved in the development of LUTS and that this urologic manifestation could be considered a socioeconomic and systemic disease with psychological etiologies. We are planning future studies to determine whether reducing levels of depression by medication would help symptoms of LUTS. This future study would highlight the role of psychological treatment in LUTS if depression exists.

An association between depression and LUTS has been reported in several cross-sectional studies (Coyne et al., 2009; Fitzgerald et al., 2007; Asplund et al., 2004; Wong et al., 2010). Furthermore, a prospective study by Hakkinen et al. (2008) reported that depressive symptoms increase the incidence of nocturia. This relationship can be explained psychologically. LUTS reduces quality of life (Coyne et al., 2009; Robertson et al., 2007) and can result in embarrassment, social anxiety, and demoralization (Breyer et al., 2014). Indeed, men with LUTS report decreased self-esteem since they perceive it as a weakness and part of the aging process (Wong et al., 2010). Nocturia may cause daytime drowsiness and decrease concentration and activity levels, all of which could lead to an increased risk for the development and progression of depression.

Several molecular pathogeneses of an association between depression and LUTS have been proposed. Steers et al. postulated that a defect in serotonin synthesis is associated with the development of depression and abnormal voiding dysfunction. Increased adrenergic tone and the hypothalamic-pituitary axis have been proposed as a mediator of depression in LUTS (Laumann et al., 2008; Steers, Litman & Rosen, 2008). Furthermore, Klausner & Steers (2004) suggested that stress-induced depression activates the corticotropin-releasing factor pathway, which functions as a mediator of emotional influences on bladder function. Moreover, inflammation represents a common mechanism in the pathogenesis of major depression and LUTS (Miller, Maletic & Raison, 2009; Johnson et al., 2010). Patients with depression frequently exhibit increased levels of C-reactive protein, tumor necrosis factor-alpha, and interleukin-6 (Miller, Maletic & Raison, 2009). Our results showed that depressed patients (PHQ-9 score ≥ 10) had higher levels of C-reactive protein (0.62 ± 1.23 mg/dL) than those without (0.40 ± 0.77 mg/dL); however, the difference was not statistically significant.

Contrary to a previous study that reported that men with greater depression scores were more likely to have LUTS (Breyer et al., 2014), our results showed that patients with the greatest depression scores were less likely to have LUTS than those with moderate depression. Breyer et al. (2014) defined the depression group as those with a PHQ-9 score ≥10. In our study, we further divided the depression group into a moderate group and a moderately severe to severe group. The moderate depression group showed higher odds with clinical LUTS (OR = 5.89, 95% CI [3.44–10.11]) and individual symptoms of LUTS, except incomplete emptying, compared to the moderately severe to severe depression group (OR = 3.74, 95% CI [1.75–8.00]). This implies that clinical LUTS may not be proportionally associated with the severity of depression. Although its pathogenesis is unclear, the aforementioned molecular pathogenesis might not be feasibly true since if molecular pathways are involved in mediating depression and LUTS, both severity levels of depression and LUTS should be proportionally elevated. This finding implies the existence of unaccounted pathogeneses that mediates LUTS.

Our results showed that there was no significant association between sleep disorders and daytime or clinical LUTS. However, a recent study reported that men with sleep disorders are significantly more likely to report both nocturia and daytime LUTS (Fantus et al., 2018). Sleep disorders increase the risk of daytime urinary symptoms including voiding and storage symptoms (Helf et al., 2011), whereas the improvement of LUTS has been associated with an improvement in sleep disorders (Helf et al., 2012). Although the proportion of men with sleep disorders was higher in the group with daytime and clinical LUTS, the sleep disorder itself might not be a factor associated with LUTS, rather a secondary feature of LUTS.

A cross-sectional study in six European countries reported that men with overactive bladder symptoms were more likely than women to report that overactive bladder symptoms had an impact on their daily work life (Irwin et al., 2006). Over 21.0% of the study population reported being worried about an interruption of meetings owing to urinary frequency, and 3.0% of the population changed or quit their jobs due to bladder control problems. This study reported a negative association of LUTS on employment issues, which is similar to our results. To our knowledge, our study is the first to report a negative association for LUTS on employment in a United States population.

This study has several limitations. First, due to the limitations of its cross-sectional design, a causal relationship could not be established. Second, measurement of the severity of LUTS was limited due to the lack of data of the International Prostate Symptom Score (IPSS). A comparison between the IPSS and PHQ-9 score may have provided more detailed information regarding the association between LUTS and depression. Third, medical conditions were assessed based on self-reporting. Therefore, some of the information for the comorbid conditions might not be accurate. Fourth, treatments for the depression and sleep disorders were not documented. Fifth, due to the retrospective analysis, the adequacy of the sample size could not be checked. Sixth, we did not consider sampling weights since our purpose was to study relations in a large community sample, rather than estimate national prevalence rates. Therefore, we did not use sampling weights in our calculations (Farmer et al., 1988). Seventh, we considered that the size of the study population was large enough to represent the US population and the values were not skewed in clinical perspective; therefore, we used geometric means and SDs and t-tests in univariable analysis. Finally, voiding diaries were not included in the NHANES datasets, which deferred the differential diagnosis of nocturnal polyuria and other causes of nocturia.

## Conclusions

Our study revealed significant associations for depression and employment statuswith clinical and daytime LUTS. The cause of LUTS is multifactorial, and psychological factors seem to be significantly associated therewith. Therefore, patients presenting with LUTS should be screened for multi-factorial etiologies using a psychological evaluation for a successful multidisciplinary treatment approach.

## Additional Information and Declarations

### Data Availability

The following information was supplied regarding data availability: NHANES datasets for 2005–2006 and 2007–2008 are available at the Centers for Disease Control and Prevention: https://wwwn.cdc.gov/nchs/nhanes/continuousnhanes/default.aspx?BeginYear=2005.
https://wwwn.cdc.gov/nchs/nhanes/continuousnhanes/default.aspx?BeginYear=2007.
